# Preprocedural imaging guiding ventricular tachycardia ablation in structural heart disease

**DOI:** 10.1002/joa3.13205

**Published:** 2024-12-19

**Authors:** Afonso Nunes‐Ferreira, Joana Brito, Nuno Cortez‐Dias, Gustavo da Lima da Silva, Fausto J. Pinto, João de Sousa

**Affiliations:** ^1^ Department of Cardiology ULSSM Lisbon Portugal; ^2^ Lisbon School of Medicine CAML, CCUL@RISE, Universidade de Lisboa Lisbon Portugal; ^3^ Cardiology Department Hospital Lusíadas Amadora Lisbon Portugal

**Keywords:** cardiac magnetic resonance, ischemic cardiomyopathy, multidetector computed tomography, nonischemic cardiomyopathy, VT ablation

## Abstract

**Background:**

Integration of preprocedural imaging techniques in ventricular tachycardia (VT) ablation may improve the identification of arrhythmogenic substrates, particularly relevant for patients with nonischemic cardiomyopathy (NICM) with sub‐optimal outcomes. We assessed the impact of advanced preprocedural imaging on the safety and long‐term efficacy of radiofrequency catheter ablation (RCA) for VT, comparing patients with NICM and ischemic cardiomyopathy (ICM).

**Methods:**

In this prospective, single‐center study, consecutive patients referred for scar‐related VT ablation underwent multidetector computed tomography (MDCT) and late gadolinium enhancement cardiac magnetic resonance (LGE‐CMR). Images were segmented with ADAS 3D software and integrated into mapping systems. Substrate map collection targeted the imaging‐predicted area of interest and the ablation aimed at eliminating all local abnormal ventricular activities. Procedural safety was evaluated with 30‐day mortality. Long‐term efficacy was assessed by survival free from appropriate ICD shocks at 36 months.

**Results:**

102 patients were included (67 ± 11 years, 94% male; 75 ICM, 27 NICM). All patients underwent MDCT and 35% also underwent LGE‐CMR. Procedural safety (4% 30‐day mortality, *p* = .95) and 36‐month efficacy were similar in both groups (88.0% vs. 74.1%, HR 2.09; *p* = .13 in ICM and NICM). Efficacy was higher in patients when VT activation mapping with VT isthmus ablation complemented substrate ablation compared to substrate‐based ablation alone (94.5% vs. 80.6%, HR 4.00; *p* < .05).

**Conclusion:**

A preprocedural imaging protocol integrated into the invasive mapping system may improve safety and long‐term efficacy, with NICM patients exhibiting outcomes comparable to those with ICM. Activation mapping of the VT on top of substrate ablation may improve prognosis.

## Graphical abstract

A structured preprocedural imaging protocol integrated into the invasive mapping system may improve both safety and long‐term efficacy, with NICM patients exhibiting outcomes comparable to those with ICM.

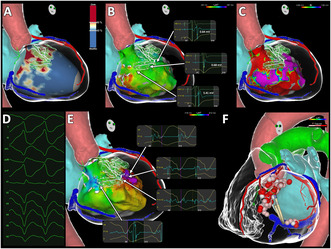

## 1 INTRODUCTION

Ventricular tachycardia (VT) ablation in structural heart disease relies heavily on accurate mapping, localization, and comprehensive understanding of the arrhythmogenic substrate to achieve optimal outcomes. This is particularly true in the context of nonischemic cardiomyopathy (NICM) where radiofrequency catheter ablation (RCA) has historically shown lower success rates compared to ischemic cardiomyopathy (ICM).^1^ In addition, traditional electroanatomic mapping techniques have limitations in visualizing structural abnormalities and scar tissue within the myocardium (e.g., septal intramural or lateral epicardial) contributing to sub‐optimal clinical outcomes with 1‐year VT‐free survival of 70%^2^ and declining to 54% at 5 years.^3^

Recently, the integration of advanced preprocedural imaging techniques has brought valuable insights into the identification and localization of arrhythmogenic substrates. Among these techniques, late gadolinium enhancement cardiac magnetic resonance (LGE‐CMR) and multidetector computed tomography (MDCT) have been studied to guide the ablation strategy, targeting specific areas. On one hand, LGE‐CMR exploits the differential distribution of gadolinium‐based contrast agents in normal and pathological tissues, providing a comprehensive and detailed assessment of scar location and morphology (scar core vs. border zone) in both ICM and NICM patients.^4^ These images can then be used to create scar maps depicting viable corridors in areas with scar, which often correlate with electrical conduction pathways within the scar (VT isthmuses). On the other hand, MDCT allows for accurate delineation of scar tissue and its distribution within the myocardium by assessing left ventricle wall thickness. Thus, it may help to delineate potential critical isthmus regions for VT.^5^ The preprocedural imaging data segmentation and further integration into the electroanatomic mapping systems may lead to a more personalized and circumscribed ablation strategy, which may improve VT ablation outcomes.

In this study we aimed to assess the impact of preprocedural imaging (MDCT and LGE‐CMR), integrated into mapping systems, on the safety and long‐term efficacy of RCA for VT, with a focus on comparing patients with NICM and ICM.

## 2 METHODS

### 2.1 Study design and population

We conducted a prospective, single‐center observational study from June 2015 to February 2021. We included all consecutive adult patients referred for scar‐related VT ablation with at least 1 episode of sustained VT or appropriate implantable cardioverter‐defibrillator (ICD) therapy and refractory to antiarrhythmic therapy, meeting the current ESC guidelines for VT ablation.^6^ VT ablation procedures were planned with MDCT and LGE‐CMR imaging, segmented using the ADAS 3D left ventricle (LV) software (ADAS3D Medical, Barcelona, Spain), and performed with 3D high‐density mapping systems as described below.

All patients provided written informed consent, the study protocol was approved by the institutional ethics committee, and it was performed in accordance with Helsinki Declaration.

### 2.2 Study objectives

The main objective of our study was to evaluate the impact of a structured preprocedural imaging strategy including MDCT and LGE‐CMR on the procedural safety and long‐term efficacy, in patients undergoing VT ablation. Specifically, we focused on comparing outcomes between patients with NICM and ICM. By integrating preprocedural imaging data into the mapping system, we sought to optimize substrate identification and localization, especially in NICM patients who historically have sub‐optimal outcomes. Safety of the procedure was evaluated by 30‐day mortality rate post‐VT ablation. Long‐term efficacy was evaluated by freedom from appropriate ICD shocks at 36 months.

Additional study objectives included evaluation of all‐cause mortality, cardiovascular (CV) hospitalization, procedural complications (defined as life‐threatening or delaying hospital discharge), VT inducibility in the end of the electrophysiology (EP) procedure, duration of the procedure, radiofrequency ablation time, and the recurrence of ICD appropriate shocks in substrate‐based ablation and substrate complemented with activation mapping ablation (addressing VT isthmus).

### 2.3 Preprocedural planning

MDCT images were obtained with delayed contrast and processed using ADAS 3D LV software, with extraction of the 3D heart anatomy and evaluation of the LV wall thickness (WT). Normal wall thickness was defined as an end‐diastolic WT >6 mm and WT ≤6 mm suggested reduced LV WT. Scar calcifications were identified and their contours were manually delineated to facilitate the generation of 3D reconstructions.

Preprocedural 3D LGE‐CMR was obtained using a 3‐T scanner (Philips Achieva 3.0 T, Quasar Dual®) in patients without cardiac implanted devices. In ICD carriers, a CMR performed previously to the ICD implant was used. CMR was not acquired in ICD carriers. MDCT was obtained in all patients using Philips CT Brilliance® (64 detectors). LGE‐CMR images were processed using ADAS 3D LV software (ADAS3D Medical, Barcelona, Spain). Full LV volume was reconstructed and divided into 9 concentrical layers from endocardium to epicardium and a 3D shell was obtained for each layer. Pixel signal intensity (PSI) maps were created and projected to each of the shells. To characterize the scar areas, a PSI‐based algorithm was applied to define the hyperenhanced area as a scar core or border zone, using 40 ± 5% and 60 ± 5% of the maximum intensity as thresholds.^7^ In these PSI maps, heterogeneous tissue channels were automatically predicted by the software, defining continuous corridors of border zone surrounded by scar core or by scar core and an anatomical barrier.

**FIGURE 1:**
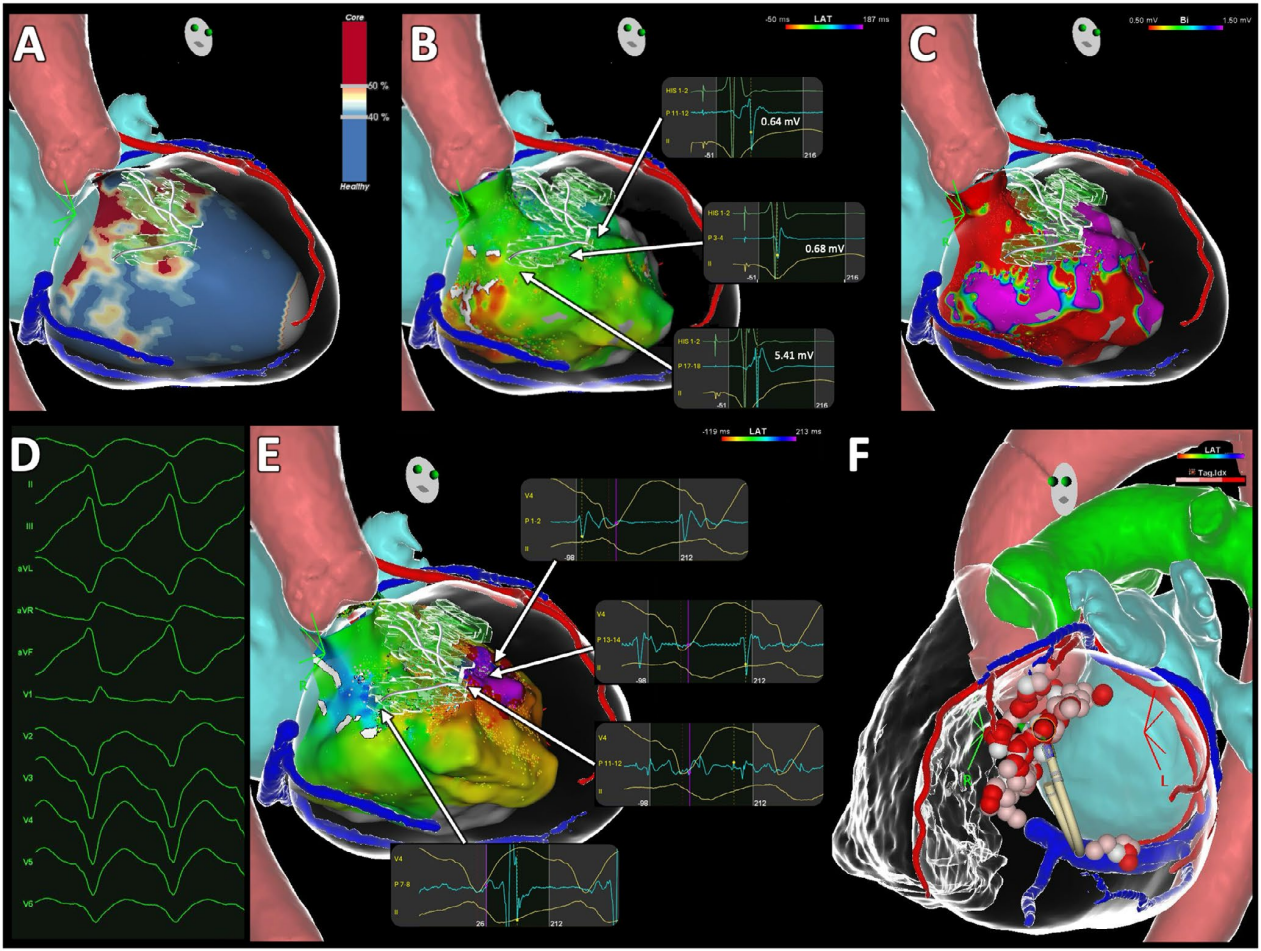
Ventricular tachycardia ablation in a 75‐year‐old male patient with nonischemic dilated cardiomyopathy. (A) Integration of the ADAS 3D reconstruction of LGE‐CMR into the Carto mapping system, revealing an intramural septal scar with several intra‐scar corridors (centerlines depicted in white, 3D channels volume represented in glass green). Remaining anatomic structures reconstructed from MDCT include left atrium (light blue), aorta (light red), left coronary artery (red), and coronary sinus (dark blue). (B) Substrate activation map collected during RV pacing, which was not particularly informative, revealing low voltage electrograms, sharp but not fragmented nor delayed, spread across the septum without areas of deceleration. (C) Substrate bipolar voltage map showing extensive scarring involving the periaortic region, the inferior wall and various septal segments, but not in the specific area where the intramural channels were predicted. (D) 12‐lead ECG of the clinical VT. (E) VT activation map revealing mid‐diastolic and pre‐systolic signals at the extremities of the intramural channels predicted by the LGE‐CMR, in accordance with a 3D intramural VT circuit. (F) Final ablation setting with unipolar RF applications on both the left and right surfaces of the ventricular septum. 3D: Tridimensional; ECG, Electrocardiogram; LGE‐CMR, Late gadolinium enhancement cardiac magnetic resonance; MDCT, Multidetector computed tomography; RF, Radiofrequency; VT, Ventricular tachycardia.

**FIGURE 2:**
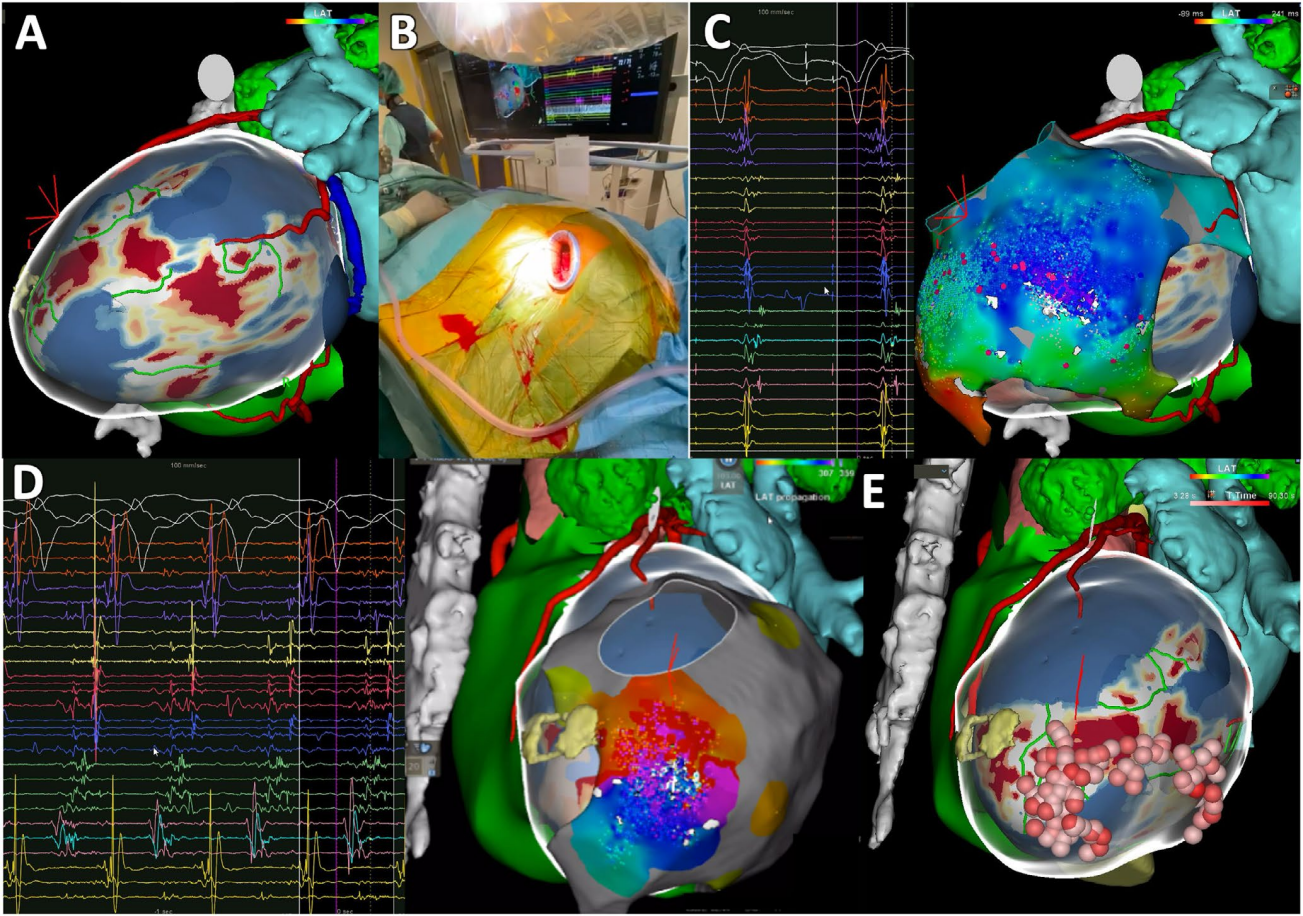
Ventricular tachycardia ablation in a 59‐year‐old male patient with valvular heart disease, with mechanical aortic and mitral prostheses, inaccessible endocardium, and extensive epicardial adhesions. As LGE‐CMR predicted arrhythmogenic channels in thin areas of the apical and mid‐ventricular segments of the lateral wall, an epicardial approach through a left lateral mini‐thoracotomy with localized manual debridement of epicardial adhesions was considered a viable treatment option. (A) ADAS 3D segmentation of LGE‐CMR depicting core scar and border zones in the apical and mid‐ventricular segments of the lateral wall, with several predicted intra‐scar corridors involving the subendocardial, mid‐mural, and subepicardial layers (green lines). Remaining anatomic structures reconstructed from MDCT include the left atrium (light blue), aorta (light red), left coronary artery (red), coronary sinus (dark blue), RV and pulmonary artery (green), and sternum (grey). (B) Left lateral mini‐thoracotomy targeting the predicted VT isthmus location. (C) Substrate activation map obtained during RV pacing with LAVA detected in the predicted area of interest. (D) VT activation map documenting the protected isthmus at a location corresponding to a predicted intra‐scar corridor. (E) Final ablation setting with unipolar RF epicardial applications. The patient remains free from arrhythmic events without medication, with a follow‐up duration now exceeding 18 months. 3D, Tridimensional; LAVA, Local abnormal ventricular activity; LGE‐CMR, Late gadolinium enhancement cardiac magnetic resonance; MDCT, Multidetector computed tomography; RF, Radiofrequency; RV, Right ventricle; VT, Ventricular tachycardia.

The 3D images reconstructed by the ADAS 3D segmented from MDCT and LGE‐MRI were exported into the invasive electroanatomical, as depicted in Figures 1 and 2.

### 2.4 Electrophysiology procedure

EP procedures were performed by 4 experienced EP physicians in the fasting state, under deep sedation or general anesthesia, with continuous invasive monitoring of arterial pressure, arterial oxygen saturation, and acid/base balance. ICD therapies were inactivated. Procedures were performed under antiarrhythmic therapy, with no prior washout of these therapies.

A quadripolar nonsteerable catheter was placed in the RV apex, and a decapolar steerable catheter in the coronary sinus if the patient had no cardiac resynchronization device. LV access was gained through an anterograde transseptal and/or a retrograde aortic approach. If clinical and/or preprocedural imaging suggested an epicardial circuit, epicardial access was performed using a Tuohy needle by the Sosa method.^8^

After LV access was obtained, a bolus of heparin was administered intravenously and repeated as necessary to achieve an activated clotting time of 250–300 s.

### 2.5 Substrate mapping

Mapping of the LV used a high‐density 3‐dimensional electroanatomical mapping (EAM) system (Carto™ system with multipolar PentaRay™ catheter, Biosense Webster; Ensite Precision with multipolar HD Grid™ catheter, Abbott; and Rhythmia™ system with multipolar IntellaMap Orion™ catheter, Boston Scientific).

Substrate mapping was directed to the areas previously identified as the most arrhythmogenic with preprocedural imaging. Maps were acquired with right ventricle (RV) pacing (quadripolar nonsteerable catheter in RV apex or with the implanted catheter in patients with an ICD or pacemaker) and/or intrinsic QRS complexes (sinus rhythm, atrial pacing, or atrial fibrillation). Maps were evaluated to identify intra‐cicatricial channels (areas of bipolar voltage <1.5 mV), in which sequential propagation of local abnormal ventricular activities (LAVAs) were observed, during or after QRS. LAVA was defined as sharp high‐frequency ventricular potentials occurring during or after the far‐field ventricular electrogram.^9^, ^10^

### 2.6 Activation mapping

After substrate mapping, VT induction was attempted by programmed electrical stimulation from the predicted protected isthmus (previously evaluated by the substrate mapping) or from the RV apex at cycle lengths of 600 or 400 ms and up to 3 extrastimuli down to the ventricular effective refractory period or 200 ms. Induced VTs were classified as clinical or nonclinical based on comparison to the 12‐lead ECG of the clinical VTs or based on the cycle length on ICD tracings. Activation map was recorded of any sustained and hemodynamically stable monomorphic VT circuits. Mapping of VT was considered complete when >90% of the tachycardia cycle length was mapped and the VT isthmus was properly delineated. The VT isthmus location was confirmed by entrainment mapping and/or pace mapping, and subsequently by response to ablation.

### 2.7 Catheter ablation and procedural endpoints

RCA was delivered with a sensor enabled open‐irrigated catheter (ThermoCool SmartTouch™ Catheter [Biosense Webster Inc.]; TactiCath™ Catheter [Abbott]; IntellaNav™ Catheter [Boston Scientific]) at 40–50 W endocardial and 30–40 W epicardial. Each application lasted at least 30 s with 10 g of force or were based on ablation index ≥550 or lesion size index ≥6.

Substrate‐based ablation aimed at the abolition of all intra‐cicatricial LAVAs located according to the imaging‐predicted area of interest from MDCT and LGE‐CMR.

After ablation, the LV was remapped, and additional substrate ablation was performed if residual LAVAs were identified. If the activation map of the sustained and hemodynamically stable monomorphic VT circuit was achieved, VT isthmus ablation was performed followed by a substrate‐based ablation. Procedural endpoints were VT noninducibility and LAVA elimination in the end of the procedure.

Venous and arterial sheaths were withdrawn after the procedure. ICD therapies were reactivated at the end of the procedure. Patients were monitored in hospital for at least 2 days. Antiarrhythmic‐drug therapy was continued according to operator discretion, ICD was implanted in patients with no previous device and patient follow‐up was performed at least every 6 months.

### 2.8 Statistical analysis

Continuous variables were presented as mean ± standard deviation or median and interquartile range for normal and non‐normal distributions, respectively. Continuous variables were compared among patient groups using the unpaired Student's t‐test or Mann–Whitney test for normal and non‐normal distributions, respectively. Categorical variables were compared among patient groups using χ^2^ tests. Cox proportional hazards model, reporting hazard ratio and 95% confidence interval (CI), evaluated freedom from appropriate ICD shocks, all‐cause mortality, and CV hospitalization. Kaplan–Meier survival analysis compared appropriate ICD shocks between ICM and NICM patients. Patients were censored at the time of death in the Kaplan–Meier analysis. For all statistical tests, *p* < .05 was considered statistically significant. All statistical analyses were performed using IBM SPSS Statistics™ version 24 (IBM, Armonk, NY).

## 3 RESULTS

### 3.1 Baseline characteristics

Between June 2015 and April 2021, 102 patients with structural heart disease were enrolled for VT ablation, 94.1% male, 66.7 ± 10.8 years of age. About 47.1% were hospitalized in electric storm, 50.0% were in NYHA functional class II and 17.6% in class III. The mean LVEF was 33.9 ± 11.3%. The etiology of structural heart disease was ICM in 75 patients (73.5%) and NICM in 27 patients (26.5%). NICM etiology was idiopathic dilated cardiomyopathy in 66.7%, postmyocarditis in 22.2%, right ventricular arrhythmogenic cardiomyopathy in 7.4% and sarcoidosis in 3.7%. Baseline characteristics are detailed in Table 1. All patients performed MDCT, and 35.3% also had LGE‐CMR imaging, processed with ADAS 3D software and integrated into the invasive mapping system. MDCT presented a 66.7 ± 48.5% sensitivity and 60.0 ± 21.2% specificity and MRI presented a 57.1 ± 53.5% sensitivity and 74.6 ± 23.0% specificity evaluating the cardiac segment of interest according to the detected LAVAs during the invasive mapping. All the patients in whom MDCT and MRI were not accurate, the region of interest was adjacent to the predicted cardiac segment.

**TABLE 1:** Characteristics of the patients at baseline.

Population characteristics	ICM group (*N* = 75)	NICM group (*N* = 27)	*p* Value
Age, years (mean ± standard deviation)	68.90 ± 9.30	60.60 ± 12.30	<.05
Male gender, *n* (%)	71.00 (94.70)	25.00 (92.60)	.69
LV EF (mean ± standard deviation), %	33.70 ± 11.00	34.80 ± 12.20	.65
NTproBNP (mean ± standard deviation), pg/mL	4338.30 ± 7253.40	3364.00 ± 3642.90	.58
Comorbidities			
Hypertension, *n* (%)	64.00 (85.30)	10.00 (37.00)	<.05
Peripheral artery disease, *n* (%)	9.00 (12.00)	0.00 (0.00)	.60
Diabetes, *n* (%)	23.00 (30.70)	6.00 (22.20)	.40
CKD (GFR < 60 mL/min/1.73m^2^), *n* (%)	39.00 (38.70)	8.00 (29.60)	.05
Obesity (BMI > 30), *n* (%)	18.00 (24.00)	5.00 (18.50)	.56
AF, *n* (%)	29.00 (37.30)	10.00 (37.00)	.88
Electric Storm, *n* (%)	30.00 (40.00)	18.00 (66.70)	.02
PAINESD (mean ± standard deviation)	13.80 ± 5.30	8.40 ± 5.60	.66
NYHA Functional Class			
I, *n* (%)	20.00 (26.70)	9.00 (33.30)	.43
II, *n* (%)	41.00 (54.70)	10.00 (37.00)	
III, *n* (%)	13.00 (17.30)	5.00 (18.50)	
IV, *n* (%)	1.00 (1.30)	3.00 (11.10)	
Medical Therapy			
ACE‐I/ARB/ARNI, *n* (%)	65.00 (86.70)	21.00 (77.80)	.48
Beta‐blocker, *n* (%)	69.00 (92.00)	25.00 (92.60)	.76
MRA, *n* (%)	45.00 (60.00)	14.00 (55.60)	.46
Amiodarone at admission, *n* (%)	56.00 (74.70)	22.00 (81.50)	.47
Beta‐blocker at admission, *n* (%)	68.00 (90.70)	25.00 (92.60)	.76
Previous ICD/CRT‐D			
Primary prevention, *n* (%)	20.00 (26.70)	13.00 (48.10)	.08
Secondary prevention, *n* (%)	39.00 (52.00)	8.00 (29.60)	

Abbreviations: ACE‐I, angiotensin‐converting enzyme inhibitor; AF, Atrial Fibrillation; ARB, angiotensin receptor blocker; ARNI, angiotensin receptor neprilysin inhibitor; CKD, Chronic Kidney Disease; CRT‐D, Cardiac Resynchronization Therapy Defibrillator; EF, Ejection Fraction; ICD, Implantable Cardioverter Defibrillator; ICM, Ischemic Cardiomyopathy; LV, Left Ventricle; MRA, mineralocorticoid receptor antagonist; NICM, Nonischemic Cardiomyopathy; NYHA, New York Heart Association.

### 3.2 Procedural safety and outcomes

Procedural safety showed no significant difference between ICM and NICM patients (30‐day mortality rate post‐VT ablation of 4% vs. 3.7%, *p* = .95, in ICM and NICM, respectively). Similarly, procedure‐related complications were comparable between ICM and NICM ablations (9.3% vs. 11.1%, *p* = .79). No predictive factors for complications were identified. Main complications are detailed in Table 2.

**TABLE 2:** Complications after EP procedure.

Procedure complications	ICM group (*N* = 75)	NICM group (*N* = 27)
Cardiac tamponade, *n* (%)	2.00 (2.33%)	0.00 (0%)
Cardiogenic shock, *n* (%)	1.00 (1.33%)	1.00 (3.7%)
Third degree atrioventricular block, *n* (%)	1.00 (1.33%)	0.00 (0%)
Pericarditis, *n* (%)	0.00 (0.00%)	2.00 (7.40%)
Aortic dissection, *n* (%)	1.00 (1.33%)	0.00 (0.00%)
Hematoma requiring transfusion, *n* (%)	1.00 (1.33%)	0.00 (0.00%)
Peripheral artery pseudoaneurysm, *n* (%)	1.00 (1.33%)	0.00 (0.00%)

Abbreviations: ICM, Ischemic Cardiomyopathy; NICM, Nonischemic Cardiomyopathy.

In the ICM group, the majority underwent an isolated endocardial procedure. Only 4 patients (5.3%) with ischemic VT required additional epicardial ablation. Conversely, a significantly higher proportion of NICM patients (22 patients, 81.5%) underwent either initial or subsequent epicardial ablation (*p* < .001).

Upon completion of the EP procedure, noninducibility of any VT was achieved in 59.6% of ICM patients (34 out of 57 assessed) and 68.4% of NICM patients (13 out of 19 assessed; *p* = .75). The procedure time was notably longer for the ICM group compared to the NICM group (276 ± 98 min vs. 127 ± 56 min, respectively; *p* < .001). However, the radiofrequency ablation duration was similar across both groups (52 ± 26 min for ICM vs. 52 ± 40 min for NICM, *p* = .91). Detailed characteristics of EP procedures are demonstrated in Table 3.

**TABLE 3:** Characterization of electrophysiological procedures.

Procedure characteristics	ICM group (*N* = 75)	NICM group (*N* = 27)	*p* Value
Preprocedural imaging with integration in ADAS 3D LV software			
LGE‐CMR, *n* (%)	27.00 (36.00)	9.00 (33.30)	.79
MDCT, *n* (%)	75.00 (100.00)	27.00 (100.00)	—
Procedural approach			
Endocardial, *n* (%)	71.00 (94.67)	5.00 (18.52)	<.01
Epicardial, *n* (%)	0.00 (0.00)	19.00 (70.37)	<.01
Endocardial‐epicardial, *n* (%)	4.00 (5.33)	3.00 (11.11)	.89
3D electroanatomic mapping system and multielectrode mapping catheter			
Carto 3 and Pentarray, *n* (%)	57.00 (76.00)	25.00 (92.60)	.18
Ensite Precision and HD Grid, *n* (%)	9.00 (12.00)	1.00 (3.70)	
Rhythmia and IntellaMap ORION, *n* (%)	9.00 (12.00)	1.00 (3.70)	
Substrate mapping			
SR or Ap/RV pacing, *n* (%)	38.00 (50.70)/37.00 (49.30)	16.00 (59.30)/11.00 (40.70)	.44
Points collected, mean ± standard deviation	2953.00 ± 2285.00	2744.00 ± 1656.00	.61
Total LV surface area, cm^2^, mean ± standard deviation	237.00 ± 82.00	225.00 ± 79.00	.95
LAVA region surface area, cm^2^, mean ± standard deviation (% total LV surface area)	22.00 ± 16.00 (9.00)	15.00 ± 9.00 (7.00)	.08
VT induction and mapping			
VT inducible, *n* (%)	62.00 (82.70)	14.00 (51.90)	<.01
Clinical VT inducible, *n* (%)	30.00 (40.00)	11.00 (40.70)	.65
Nonclinical VT inducible	46.00 (61.30)	3.00 (14.80)	.54
Activation mapping, *n* (%)	26.00 (34.70)	9.00 (33.30)	.87
Pace mapping, *n* (%)	20.00 (26.70)	5.00 (18.50)	.61
Procedure data			
Total radiofrequency energy delivery time, min	51.60 ± 26.30	52.40 ± 40.40	.91
Total x‐ray exposure, min	26.20 ± 17.40	29.70 ± 16.20	.36
Procedure endpoints			
Assessment of VT inducibility, *n* (%)	57.00 (74.70)	19.00 (70.40)	.75
Clinical VT still inducible, *n* (%)	1.00 (1.30)	0.00 (0.00)	.57
Nonclinical VT still inducible, *n* (%)	21.00 (28.00)	5.00 (18.50)	.48
Noninducibility of any VT, *n* (%)	35.00 (46.70)	14.00 (51.90)	.45
Elimination of all late potentials, *n* (%)	66.00 (88.00)	23.00 (85.20)	.41

Abbreviations: 3D, three‐dimension; Ap, atrial pacing; IQR, interquartile range; LAVA, local abnormal ventricular activity; LGE‐CMR, late gadolinium enhancement cardiac magnetic resonance; LV, left ventricle; MDCT, multidetector computed tomography; n, number; SR, sinus rhythm; RV, right ventricle; VT, ventricular tachycardia.

### 3.3 Long‐term efficacy outcomes

During a median follow‐up of 29.1 (IQR 14.2–48.8) months, out of the 102 patients, 32 (31.4%) died (34.4% because of cardiovascular causes), 20 (19.6%) received appropriate ICD shocks, and 33 (32.4%) were hospitalized because of cardiovascular causes.

**FIGURE 3:**
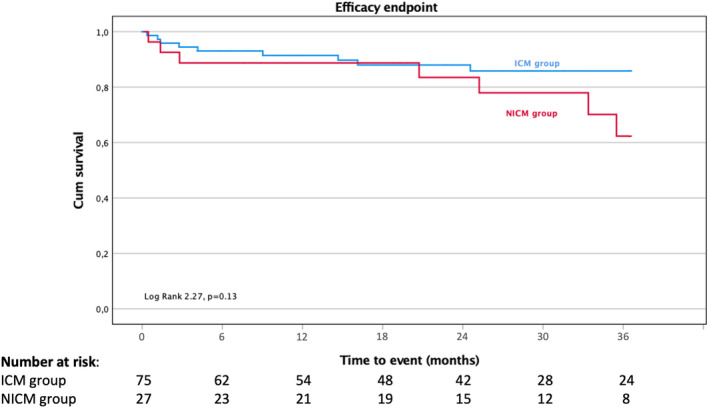
Long‐term efficacy, assessed by the cumulative survival free from appropriate ICD shocks. ICM, Ischemic Cardiomyopathy; NICM, Nonischemic Cardiomyopathy.

The 36‐month efficacy was comparable between ICM and NICM patients, 88% (66/75) versus 74.1% (20/27), respectively (*p* = .13), as illustrated in Figure 3. No predictive factors for the primary outcome were identified. Antiarrhythmic therapy was discontinued in 70.7% in ICM and in 66.7% in NICM after ablation (*p* = .60).

In a subgroup analysis regarding exclusive endocardial procedures and epicardial procedures (7 procedures of which with endo‐epi approach), there were no differences in 36‐month efficacy 84.2% versus 84.6% (*p* = .84).

Furthermore, the rates of freedom from all‐cause mortality (78.5% [59/75] vs. 66.7% [18/27], *p* = .22) and CV hospitalizations (73.3% [55/75] vs. 66.7% [18/27], *p* = .42) did not differ significantly between the two groups.

### 3.4 Substrate complemented with VT activation mapping and isthmus ablation

In 34.3% of patients (35/102), the clinical VT was inducible and mappable, with similar rates in ICM (34.7%) and NICM (33.3%), *p* = .87. Long‐term efficacy was significantly higher when VT activation mapping and VT isthmus ablation were used in conjunction with substrate‐based ablation, compared to substrate‐based ablation alone (94.5% [33/35] vs. 80.6% [54/67], HR 4.00 95% CI [1.21–19.89], *p* < .05). In patients where VT activation map could not be performed, the critical isthmus predicted using preprocedural imaging enabled comparable outcomes to those with substrate and VT activation mapping (LogRank 1.47, *p* = .23, supplementary material Figures S2 and S3). Moreover, the rates of freedom from all‐cause mortality and CV hospitalization did not differ significantly between the groups (HR 0.62, 95% CI [0.24–1.54], *p* = .29 and HR 0.71, 95% CI [0.32–1.53], *p* = .37, respectively).

## 4 DISCUSSION

The main findings from this prospective study, which involved patients undergoing scar‐related VT ablation after a structured preprocedural imaging evaluation using LGE‐CMR and/or MDCT, are as follows:

- Preprocedural imaging with MDCT and LGE‐CMR, complemented by ADAS 3D segmentation and integration into the EAM system, is not only feasible and safe but it also potentially enhances VT ablation outcomes in NICM patients.
- The safety profile of ablation was favorable in both ICM and NICM patients, with comparable 30‐day mortality rates postablation (4% vs. 3.7%, *p* = .95) and similar rates of procedural‐related complications (9.3% vs. 11.1%, *p* = .79).
- 36‐month efficacy measured as freedom from appropriate ICD shocks was comparable for both ICM and NICM patients (88.0% vs. 74.1%, *p* = .13) using preprocedural planning.
- The addition of activation mapping to substrate mapping in VT ablation significantly improved long‐term efficacy compared to substrate‐based ablation alone (94.5% vs. 80.6%, *p* < .05) for ICM and NICM groups.

Ventricular tachycardia in structural heart disease significantly impacts hospitalization rates, long‐term survival^11^, ^12^, ^13^ and quality of life.^14^ With the broadening indications and the development of new techniques, RCA's use has increased for VT patients with structural heart disease. Previous studies on VT ablation outcomes in NICM patients are limited and have raised safety concerns, often showing poorer outcomes compared to ICM patients.^1^ These modest results have led to a deferred approach to ablation in NICM patients, typically at a more advanced disease stage. The complexity of circuits, including a higher rate of intramural and epicardial scarring, may contribute to these safety concerns and poorer outcomes in NICM patients.

Advancements in MDCT and LGE‐CMR imaging have significantly improved the accuracy of identifying fibrotic tissue, localizing arrhythmogenic substrates, and targeting VT ablation procedures.^15^, ^16^ The use of ADAS 3D LV software for processing these images can predict VT channels in patients with LGE‐CMR and allows for a detailed evaluation of wall thickness, serving as a surrogate marker to predict scar‐related VT, especially in patients with transmural scars.^17^ By incorporating processed LGE‐CMR/MDCT with ADAS 3D into our preprocedural workflow, we demonstrated similar safety and 36‐month efficacy endpoints in both ICM and NICM patients. Preprocedural imaging revealed a more complex 3D arrhythmic substrate, leading to a higher rate of epicardial approach in NICM patients (5.3% in ICM vs. 81.5% in NICM, *p* < .001). This data collection before the EP procedure was pivotal in selecting the appropriate access (endo, epi, or endo‐epi) and optimizing the timing of intra‐procedure heparin, mainly after an epicardial access was established. Moreover, the ADAS 3D software accurately predicted most VT channels, directing mapping to the areas of interest and tailoring ablation strategies to more personalized and confined regions, potentially reducing procedure and general anesthesia duration.

Dinov et al. reported lower VT‐free survival rates in NICM patients compared to ICM patients at 12 months (57% vs. 40.5%, *p* = .01).^1^ In our study, we observed comparable rates of freedom from appropriate ICD shocks in both ICM and NICM patients over a 36‐month follow‐up (88% vs. 74.1%, *p* = .14). We hypothesize that the safety and efficacy in NICM patients may have been enhanced by employing a structured preprocedural imaging protocol. This positive trend should prompt the consideration of such protocols in the treatment of NICM patients, potentially bringing their outcomes more in line with those of ICM patients. Our approach signifies a shift from a one‐size‐fits‐all ablation strategy towards a more precise and individualized approach.

It is important to note that many patients undergoing VT ablation have ICDs, which can introduce artifacts in CMR imaging. To mitigate this, the use of wideband CMR sequences has been shown to reduce artifacts while preserving the accuracy of substrate characterization.^18^ Our study utilized 3 T CMR imaging, limiting CMR to patients without cardiovascular implantable electronic devices (CIEDs). For those patients requiring CIEDs and identified as high‐risk of VT, CMR was performed prior to device implantation. This strategic adjustment in our imaging protocol reflects a proactive approach to the evolving needs of patients with CIEDs, ensuring the ongoing requirements of ablation strategies. The integration of wideband sequences holds the potential to further enhance our results.

We observed a significant improvement in long‐term efficacy outcomes when VT activation mapping and VT isthmus ablation were added to substrate ablation, compared to substrate‐based ablation alone (94.5% vs. 80.6%, *p* < .05). These results suggest a prognostic benefit of mapping clinical VT to refine ablation strategies and reduce VT recurrence. However, VT could only be mapped in 34.3% of our patients. To achieve similar results in patients without VT mapping, we demonstrated that preprocedural planning with VT protected isthmus prediction, complementing substrate‐based ablation, presents comparable efficacy outcomes. This underscores the importance of preprocedural imaging in enhancing traditional ablation methods.

Preprocedural imaging is crucial for identifying cardiac scar tissue and arrhythmogenic areas within the scar. Nonetheless, it is common to encounter multiple regions of interest, presenting a challenge to address them all comprehensively. The analysis of the VT ECG and, when possible, the activation map of the clinical VT are beneficial to prioritize ablation targets. Considering the frequency of nontolerated VT in EP studies, it may be relevant to implement techniques that enable activation mapping of short‐duration or unstable VT to complement substrate‐based ablation. Recent advancements in noninvasive 3D mapping systems may overcome these challenges by rapidly mapping unstable or short‐lived VTs,^19^ offering the potential for further optimization of preprocedural planning.

## 5 LIMITATIONS

The potential under‐representation of certain NICM etiologies, such as sarcoidosis or arrhythmogenic RV cardiomyopathy, which historically have a worse prognosis, might lead to an overestimation of our outcomes. Furthermore, the limited number of patients may underpower the study to detect a significant difference between ICM and NICM patients. The fact that all patients performed preprocedural imaging to guide ablation may limit the direct impact of preprocedural imaging in efficacy, even though none of the variables we analyzed were predictive factors for our outcome. Additionally, the absence of 1.5 T wideband CMR sequences could contribute to an underestimation of our results. Lastly, the reliance on ICD shocks to assess VT recurrence may not fully capture nonsustained VT events or VT that is converted with antitachycardia pacing.

## 6 CONCLUSIONS

This prospective study of patients referred for scar‐related VT ablation demonstrates that the integration of a structured preprocedural imaging protocol into the invasive mapping system is feasible and potentially enhances safety and long‐term efficacy. Notably, NICM patients showed outcomes comparable to those with ICM. Furthermore, activation mapping of the clinical ventricular tachycardia on top of substrate ablation seems to improve prognosis.

## FUNDING INFORMATION

This study was partially funded by Associação Lusíadas Knowledge Center.

## CONFLICT OF INTEREST STATEMENT

Drs. Nuno Cortez‐Dias and João de Sousa received travel and consulting fees from Biosense Webster, Boston Scientific, and Abbott Medical.

## ETHICS STATEMENT

The study protocol was approved by the institutional ethics committee. All patients provided written informed consent.

## CLINICAL TRIAL REGISTRATION

N/A.

## Supporting information


Data S1.
