# Supplementary material for: Preprocedural imaging guiding ventricular tachycardia ablation in structural heart disease
Source: J Arrhythm. 2024 Dec 19;41(1):e13205. doi: 10.1002/joa3.13205 (PMC11730700; doi:10.1002/joa3.13205)

***Supplementary material***

*Figure 1.*

Ventricular tachycardia ablation in a 57-year-old male patient with a dilated cardiomyopathy and recurrent VT refractory to amiodarone. A. ADAS 3D segmentation of LGE-CMR depicting core scar and border zone in epicardial region of segment 11 with a predicted intra-scar corridor. Remaining anatomic structures were segmented from cardiac CT (left atrium in blue, pulmonary artery in green, coronary arteries in red); B. Substrate map obtained during RV pacing with LAVA at the predicted area of interest; C. Clinical VT activation mapping with protected isthmus colocalized at the LGE-CMR predicted corridor; D. Final ablation setting.


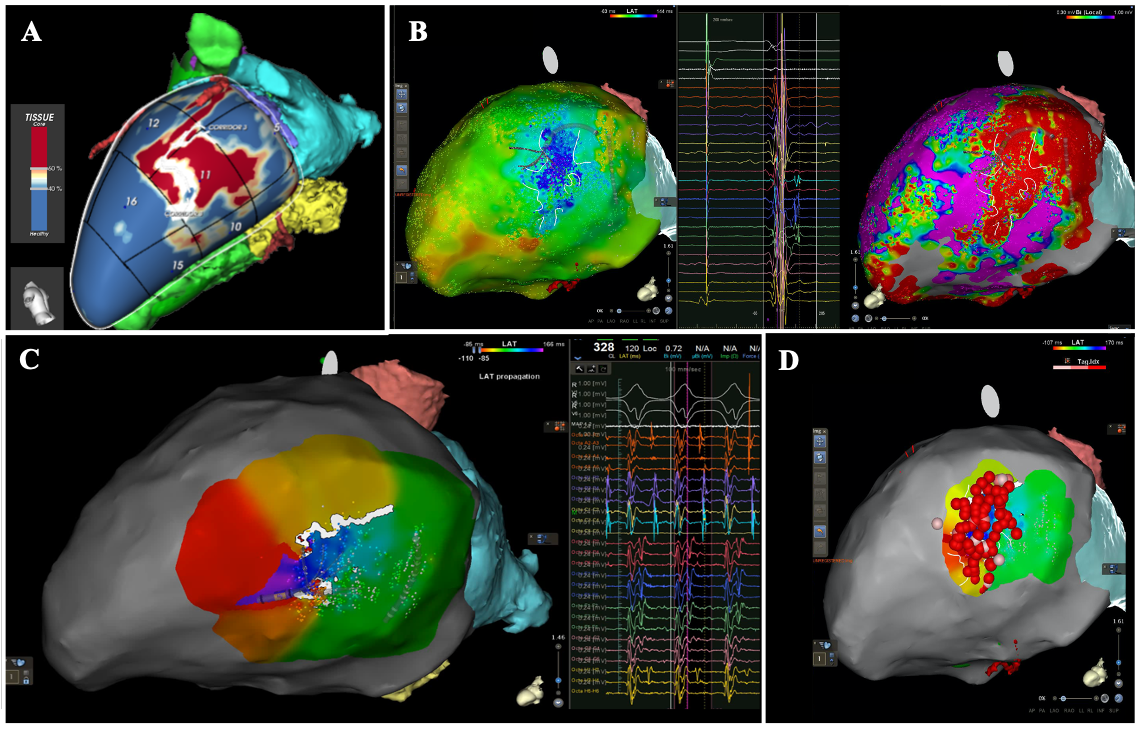


Figure 2. Long-term efficacy, assessed by the cumulative survival free from appropriate ICD shocks in subgroup analysis.


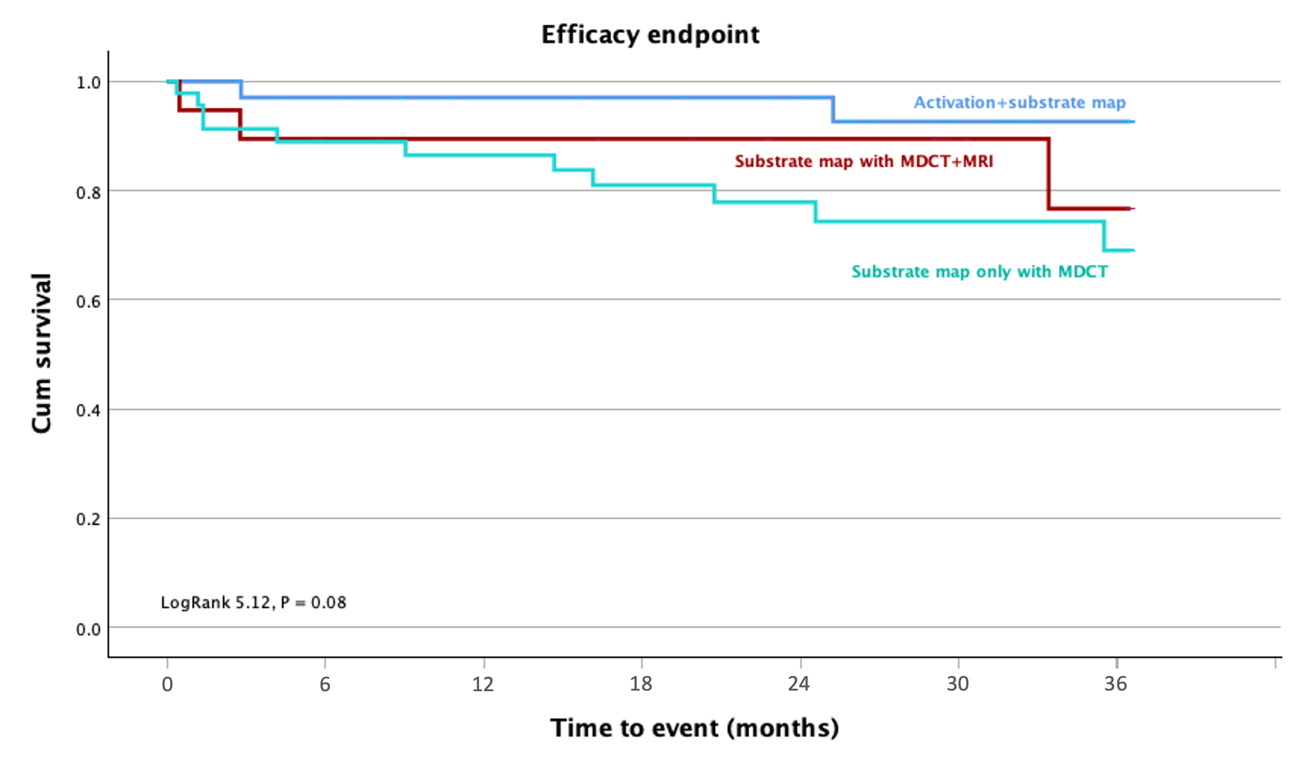


Figure 3. Long-term efficacy, assessed by the cumulative survival free from appropriate ICD shocks in subgroup analysis.


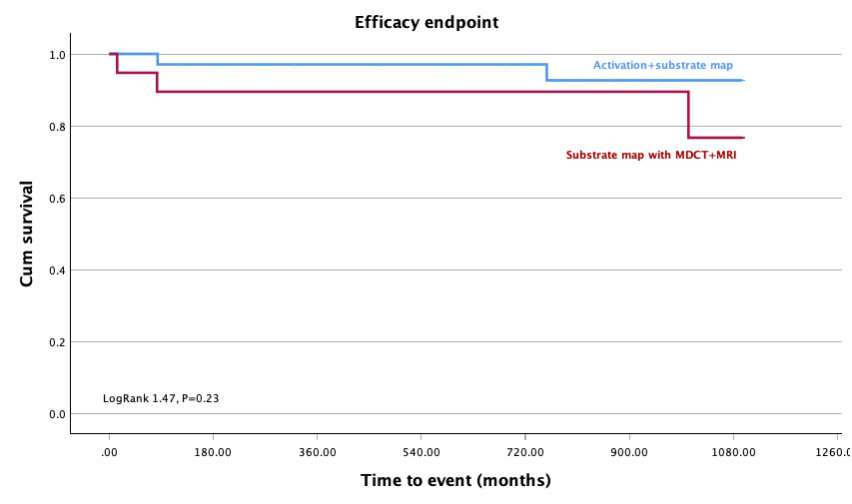

Supplement: Supplementary file 1 — Data S1. [file JOA3-41-e13205-s001.docx]
